# Resident Education in Principles and Technique of Bowel Surgery Using an Ex-Vivo Porcine Model

**DOI:** 10.1155/2010/852647

**Published:** 2010-03-22

**Authors:** M. Bijoy Thomas, V. Dandolu, P. Caputo, R. Milner, E. Hernandez

**Affiliations:** ^1^Department of Obstetrics and Gynecology, Temple University School of Medicine, Philadelphia, 19104 PA, USA; ^2^Temple University School of Medicine, Philadelphia, 19104 PA, USA; ^3^Institute for Clinical Simulation and Patient Safety, Temple University School of Medicine, Philadelphia, 19104 PA, USA

## Abstract

*Objective*. improve competency of residents with lysis of adhesion (LOA) and bowel surgery using a porcine model. *Study Design*. Pig bowel was removed at time of an anatomy laboratory, cleansed, and used to demonstrate surgical techniques and principles of LOA, repair of enterotomy, bowel resection, and anastomosis. Participants were surveyed pre- and posttraining session using 10 point Likert scale. *Results*. Thirty one residents at varying levels of training participated. After the training session, there was a significant improvement noted in mean scores for comfort level with LOA (6.3 versus 7.7, *P* = .007), comfort level with enterotomy repair (2.8 versus 6.4, *P* < .0001), understanding principles of LOA (5.0 versus 7.7, *P* < .0001), understanding principles of enterotomy repair (3.5 versus 7.0, *P* < .0001), and familiarity with instruments used (5.8 versus 7.3, *P* = .01). *Conclusion*. Training sessions using ex-vivo porcine model improve resident perception of knowledge and comfort with LOA and enterotomy repair.

## 1. Introduction

Extensive adhesions are frequently encountered during gynecological surgery secondary to endometriosis, pelvic inflammatory disease, and multiple pelvic surgeries. Obstetrics and Gynecology (OB GYN) residents should be well trained in complex surgical skills such as lysis of adhesion (LOA) as well as repair of inadvertent enterotomy. The operating room provides a suboptimal environment to learn complex surgical skills such as bowel surgery or LOA. This is secondary to patient safety issues, time and fiscal constraints, trainee anxiety, and significant variation in patients. Hence it has been recommended, that a large part of teaching and practice should occur outside the operating room [1]. 

In a recent editorial Goff points out, that the keys to learning surgical skills are step-by-step demonstration, repetition, ability to make mistakes, and a lack of time constraint [2]. The old school approach to learning surgical skills involved prolonged observation by the trainee, followed by repeated mentored performance of the procedure on patients. Multiple studies conducted during general surgery and obstetrics and gynecology residencies have shown that laboratory-based training improves technical skills and competency when subsequently performing the procedures on actual patients [1, 3–6]. “Hands on” training on cadaver and model simulators has been shown to be superior to standard training and didactics [3, 7–9]. Laboratory-based training allows trainees to learn in a low-stress environment where mistakes are permissible, procedures can be repeated multiple times to improve muscle memory, and informative feedback can more rapidly lead to skill competency [1]. 

We sought to improve competency of OB GYN residents with LOA and bowel surgery using a porcine model. The objective of our study was to conduct a pilot study to assess the value of an ex-vivo porcine bowel model in resident education. 

A porcine model was selected because of similarities in anatomic appearance to human bowel. The stomach and small bowel in pigs and humans are very similar in appearance. Large intestine on the other hand has a spiral configuration in the porcine bowel [10]. There are extensive amounts of native adhesions in the porcine bowel, which makes it a good model to practice LOA. Easy availability and affordable cost are other advantages of using a porcine bowel model for resident education.

## 2. Materials and Methods

**Figure 1:**
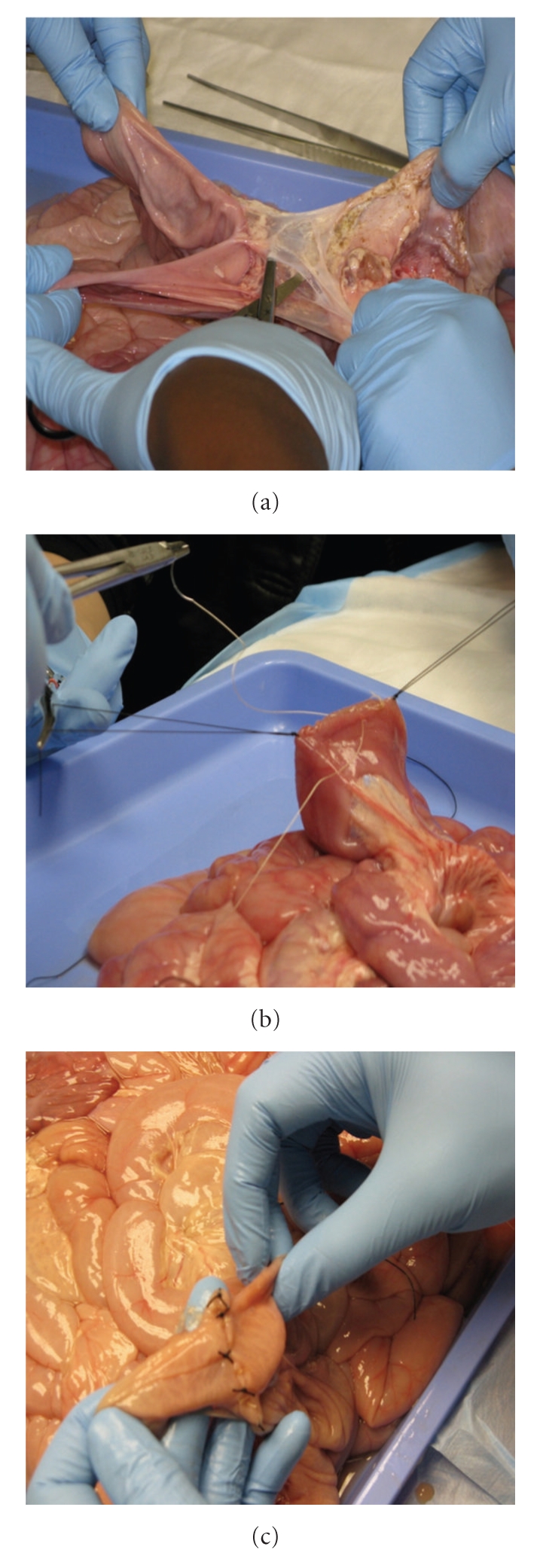
Bowel Surgery using ex-vivo Porcine Bowel Model. (a) Lysis of adhesions; (b) Enterotomy repair; (c) Bowel resection and anastomosis.

The simulation experiment was conducted at the university Institute for Clinical Simulation and Patient Safety. Porcine small intestines were harvested from euthanized pigs previously procured by the Department of Surgery for an Institutional Animal Care and Use Committee (IACUC) approved resident education animal laboratory. Pig bowel was removed in its entirety with its mesentery at the time of a surgical residents' education animate laboratory session. The bowel was cleaned by securing one end of the bowel to a piece of tubing attached to a sink faucet and flushing through the other open end with water until all contents were removed. Sections were then drained, patted dry, splayed out flat in a zip-lock bag and frozen at −20°C until needed. Bagged specimens were moved to a refrigerator (4°C) 18–24 hours prior to the laboratory session to allow them to slowly thaw. Bowel sections were removed from the zip-lock bags and placed on absorbent underpads for the training sessions. The model was then used to demonstrate surgical techniques and principles of LOA, repair of incidental enterotomy, and bowel resection and anastomosis (Figures 1(a), 1(b), and 1(c)). The surgical instrument set and the suture material were the same as the ones used in the operating room.

## 3. Results

**Figure 2:**
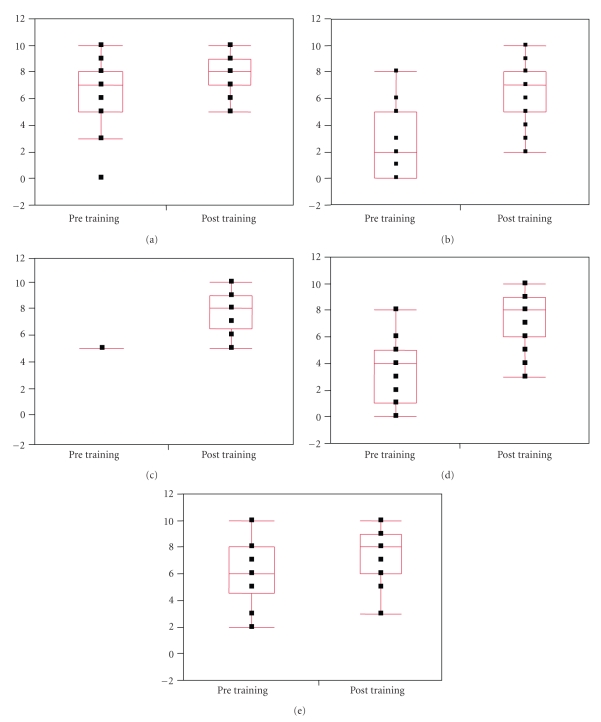
Quantile Box Plots schematically demonstrating pre and post session surveys obtained from residents completing the training session. (a) Comfort level with lysis of adhesion (Mean Scores: 6.3 versus 7.7, P = .007); (b) Comfort level with enterotomy repair (Mean Scores: 2.8 versus 6.4, P < .0001); (c) Knowledge of principles of lysis of adhesion (Mean Scores: 5.0 versus 7.7, P < .0001); (d) Knowledge of principles of enterotomy repair (Mean Scores: 3.5 versus 7.0, P < .0001); (e) Familiarity with instruments used with bowel surgery (Mean Scores: 5.8 versus 7.3, P = .01).

31 OB GYN residents participated in the training session on LOA and enterotomy repair using the porcine model. Three stations were set up. Groups of 5-6 residents practiced at one of the stations under direct supervision of faculty gynecologic oncologists for a period of 1 hour. The residents then practiced on their own at one of the two stations for an additional period of 1-2 hours, with each resident spending at least 20 minutes as the primary surgeon. A participant self-assessment questionnaire was used to assess the effect of the training session. Pre and post-training surveys were obtained from the residents in three areas: Figure 1(a) comfort level with LOA, Figure 1(b) comfort level with enterotomy repair, Figure 2(a) knowledge of principles of LOA, Figure 2(b) knowledge of principles of enterotomy repair, and (3) familiarity with instruments used for intestinal surgery implies working with GI staplers and technical aspect of bowel surgery. The Likert Scale is a subjective scale, which a participant uses to quantify his or her experience. A 10 point Likert Scale was used to collect data with 0 being no confidence and 10 being utmost confidence, and the mean score for each question was used as the main outcome measure. The data collected was analyzed using the paired student's *t* test. 

The level of training of the participating residents was as follows: PGY 1, 7 (23%); PGY 2, 8 (26%); PGY 3, 11 (35%); PGY 4, 5 (16%). Prior experience with extensive LOA was reported as none for 2 (6%), <5 cases for 5 (16%), 5 to 15 cases for 10 (32%), >15 cases for 14 (45%) residents. Prior experience with enterotomy repair was reported as none for 10 (32%), <5 cases for 17 (55%), and 5 to 15 cases for 4 (13%) residents. 

A significant improvement was noted in all parameters of the training survey. Quantile box plots schematically demonstrating pre and post session surveys obtained from residents completing the training session are shown in Figure 2. The box plot surrounds the middle half of the data distribution and the lines extending from the box show the tails of the distribution. After the training session, there was a significant improvement noted in mean scores for the comfort level with LOA (6.3 versus 7.7, *P* = .007) (Figure 2(a)), and comfort level with enterotomy repair (2.8 versus 6.4, *P* < .0001) (Figure 2(b)). Significant improvement was also noted in mean scores after training session in knowledge of principles of LOA (5.0 versus 7.7, *P* < .0001) (Figure 2(c)) and principles of enterotomy repair (3.5 versus 7.0, *P* < .0001) (Figure 2(d)). There was improvement noted in familiarity with the instruments used for intestinal surgery (5.8 versus 7.3, *P* = .01) (Figure 2(e)). 

On stratifying the data, the training session was more helpful for junior residents (PGY1, PGY2). After the training session, there was a more significant improvement noted in junior residents in mean scores for the comfort level with LOA (junior 6.1 versus 7.7, *P* = .01/senior 6.6 versus 7.7, *P* = .01), comfort level with enterotomy repair (junior 1.9 versus 6.4, *P* < .001/senior 3.7 versus 6.4, *P* < .001), in principles of enterotomy repair (junior 3.0 versus 7.0, *P* < .001/senior 4.1 versus 7.0, *P* < .001),and familiarity with the instruments used for intestinal surgery (junior 5.6 versus 7.3, *P* = .01/senior 6.1 versus 7.3, *P* = .05). 

The expense report for the entire project was $1300. Porcine small intestines were harvested as byproducts of resident education animal laboratory at no cost. We have procured porcine small intestine from a local meat supplier for a price of $10 for other similar projects. The cost related to utilizing the facility, the technical support for the retrieval and preparation of the porcine bowel model, and the provision of basic laparotomy surgical instrument set was $600. The cost of suture material (3–0 vicryl, 3–0 silk) utilized was $100. The instructor fee for the training session was $600.

## 4. Comment

Extensive adhesions are frequently encountered during gynecological surgery and residents need to be well versed with LOA as well as repair of inadvertent enterotomy. We sought to improve competency of obstetric and gynecologic residents with LOA and bowel surgery, in a pilot study, using an ex-vivo porcine model. 31 residents at varying levels of training participated in our training session. Prior experience with extensive LOA and enterotomy repair varied as well. The present study suggests that training sessions on a porcine model improves resident perception of technical knowledge and comfort level with LOA and enterotomy repair and familiarity with instruments used for intestinal surgery.

Simulation has been widely used in the military, the airline industry, and now in medical specialties. Simulation attempts to recreate scenes to teach, test, and prepare for a particular scenario one may encounter [11]. The goal of any surgical simulator based training is to help the trainee acquire and refine the cognitive and technical skills necessary to perform a surgical procedure [12]. Surgical simulation should sustain deliberate practice in a safe environment, provide access to expert tutors, map real life clinical experiences, and provide a trainee-centered milieu that is constructive to learning [12]. Cadaver training and model simulators have been shown in multiple studies to be superior to standard training alone, when subsequently performing the procedures on actual patients [3, 6, 8, 9].

There are 2 types of surgical simulators. The low fidelity simulators use material and equipment that are less similar to the true surgical environment. Low fidelity models sacrifice realism for portability, lower cost, and potential for repetition. Examples of low fidelity simulators include bench models such as video box trainers, knot tying boards, and episiotomy repair models. The high fidelity simulator provides realism through characteristics such as visual cues, tactile features, and feedback capabilities. Examples of high fidelity simulators would include virtual reality simulators, procedural simulators, and live animal models. Live animal model are considered to be of high fidelity and are most desirable for complex skills. Drawbacks to these models are high cost, limited availability, and moral and ethical concerns [12]. 

Our model would be considered as an intermediate to a high fidelity surgical simulator for training residents in LOA and enterotomy repair. The porcine model was selected because of similarities in the anatomic appearance to human bowel especially the stomach and small bowel [10]. There are also extensive amounts of adhesions in the porcine bowel, which makes it a good model to practice LOA. The pig bowel, along with the entire mesentery, can be retrieved at the time of an anatomy or surgical animal laboratory, or can be purchased from a local meat supplier. The retrieval, cleaning, and setting up the training session is relatively simple and straightforward. 

However, unlike other high fidelity models, our model is inexpensive. The expense report for our entire project was $1300 and allowed for participation of 31 residents for 5-6 hours. The cost of surgical simulation systems can be anywhere from $4,000 to $200,000 [3, 8]. Pig surgical laboratories cost approximately $2000 to train 6 residents for 4 hours [1]. In an era of shrinking budgets, easy availability and affordable cost are advantages to using an ex-vivo porcine bowel model for resident education. Several studies have shown that inexpensive training models are as good as or better than “high tech” expensive models. Grober et al. conducted outcome analysis of vasovasostomy in junior residents randomized to high fidelity model training (live rat vas deferens; *n* = 21); low fidelity model training (silicone tubing; *n* = 19); or didactic training alone (*n* = 10). Surgical skills' training on low fidelity bench models was as effective as high fidelity model training for the acquisition of technical skill among novice surgeons. Both low and high fidelity bench model training were superior to didactic training [7]. Other authors have reported similar studies showing equal effectiveness of low and high fidelity surgical models [8, 9, 13]. 

The present study is a pilot study to assess the value of an ex-vivo porcine bowel model in resident education. Limitations of the study are the small sample size, subjective survey, and lack of objective evidence of improved surgical performance. 

There are no validated instruments in the literature specific to assessing the knowledge and skill level of bowel surgery. In this study self-assessment questionnaire pre and post intervention was used to assess effect of the training session. Likert scale was used to collect responses and the mean score for each question was used as the main outcome measure. Mandel et al., has noted a strong correlation between resident self-assessment and faculty assessment after a surgical bench training session [14]. This study however lacks objective and valid instruments to measure the specific technical skills involved in bowel surgery. The Objective Structured Assessment of Technical Skills (OSATS) which uses task specific checklists, global rating scales, and overall pass/fail judgment is a valid, objective, and reliable method to asses surgical skills [15, 16]. We plan to develop a specific OSATS for bowel surgery and LOA in the future and use independent evaluator to assess the participant skill level. We plan to collaborate with other teaching programs and have “blinded” evaluators assess resident performance in the laboratory and operating room setting using a task specific checklists, global rating scales, and overall pass/fail judgment. 

The ultimate purpose of any surgical simulation is to help surgeons reach a skill level that will translate into improved surgical performance in the operating room. The current study does not address this issue. There is a potential for transmission of infectious disease with the porcine model, hence appropriate safety precaution are necessary. 

Any surgical simulator should be considered only as a complementary tool to accelerate learning and not as a replacement for actual patient encounter which is the corner stone of medical education [12]. Sutherland et al. demonstrated this in a systemic review of 30 randomized controlled trials of surgical simulation. The author concluded that none of the methods of simulator-based training was superior to formal surgical training [3]. 

Despite the limitations, our pilot study suggests that the porcine bowel model is a good teaching model that utilizes modest resources. Identifying more effective methods to teach and assess surgical skills will benefit not only our trainees but also the patients for whom we care [1, 5]. In conclusion, training sessions on an ex-vivo porcine model improves resident perception of their technical knowledge and comfort level with LOA and enterotomy repair. This is an inexpensive, safe, and efficient way to teach these surgical skills. We plan to develop a specific OSATS for bowel surgery and LOA.
